# Bedbugs and Healthcare-associated Dermatitis, France

**DOI:** 10.3201/eid1506.081480

**Published:** 2009-06

**Authors:** Pascal Delaunay, Véronique Blanc, Marc Dandine, Pascal Del Giudice, Michel Franc, Christelle Pomares-Estran, Pierre Marty, Olivier Chosidow

**Affiliations:** Centre Hospitalier Universitaire de Nice, Nice, France (P. Delaunay, C. Pomares-Estran, P. Marty); Centre Hospitalier d’Antibes–Juan les Pins, Antibes–Juan les Pins, France (V. Blanc); Maison de Retraite du Centre Hospitalier Pierre-Nouveau, Cannes, France (M. Dandine); École Nationale Vétérinaire, Toulouse, France (M. Franc); Centre Hospitalier Intercommunale de Fréjus/Saint-Raphaël, Fréjus, France (P. Del Giudice); Université Pierre-et-Marie-Curie–Paris 6, Paris, France (O. Chosidow)

**Keywords:** Bedbugs, Cimex lectularius, healthcare, dermatitis, nursing home, hospital outbreak, parasites, France, letter

**To the Editor:** Bedbugs (*Cimex lectularius*) are hematophagous insects. Adults are 4–6 mm long, flattened, oval and wingless, and brown to brownish-red (Figure, panel A) (*1*). They may feed in the wild on birds or bats (*2*), but they are mainly associated with human dwellings and can be found on furniture and clothing (*3*). Because bedbugs are nocturnal and feed painlessly only in the dark, while humans sleep, initial bedbug proliferation usually goes unnoticed until several weeks later when the patient discovers a pruritic cutaneous eruption of unknown origin (*4*). Decades ago, bedbugs were frequently found worldwide, but reports of cases in industrialized countries have progressively declined, probably the result of improved living conditions (*3*). They nonetheless remain a pest in less-developed countries and in the wild (*5*). The past 10 years have seen the revival of this insect in industrialized countries (*3*,*6*,*7*). Increasing reports describe isolated cases or bedbugs spreading throughout a single building (*8*). We report an outbreak of healthcare-associated dermatitis caused by bedbugs in a hospital nursing home in Cannes, French Riviera.

**Figure:**
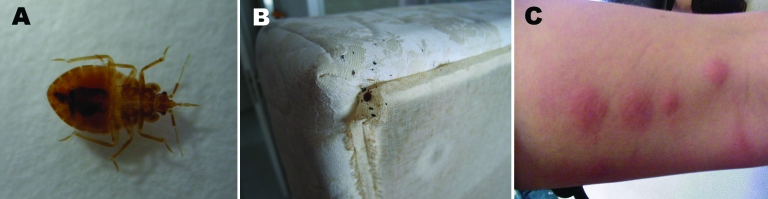
A) Adult bedbug (*Cimex lectularius*); B) mattress infested with bedbugs (an adult, eggs, and dejecta); C) dermatitis caused by bedbug bites.

In July 2007, Mrs. Q arrived, with her bed and mattress, for admission to a single room in a hospital nursing home. This facility has 112 rooms located on 2 floors, each having A and B wings. Mrs. Q’s first lesions, diagnosed as insect bites, appeared in October 2007. Concomitantly, Mrs. T, a long-term resident of the room across the hall (1.5 m away), developed similar lesions. Examination of Mrs. Q’s room led to the discovery of an aggregation of 200 *C. lectularius* bedbugs beneath her mattress. In Mrs. T’s room, 15 bedbugs were identified (Figure, panel B). Suspected insect excreta were also found in another nearby room. A private company conducted a nonspecific pest-control intervention in these 3 rooms.

In November 2007, another 2 residents in rooms located 3 and 6 m away from Mrs. Q’s had insect-bite dermatitis: 15 bedbugs were found in each room. Over a 3-week period, the nursing home staff performed the second pest-control intervention in these 2 infested rooms and also treated 10 adjacent rooms. They disassembled furniture and applied insecticides to furniture, room corners (imiprothrine and cypermethrine), and clothing (esdepallethrine and piperonyl butoxide).

No additional skin lesions occurred during the next 4 months, and no new resident was admitted. In March 2008, a new long-term resident developed similar bedbug-dermatitis lesions (Figure, panel C); 12 *C. lectularius* bedbugs were found in his room (33 m from Mrs. Q’s room, same floor, wing B). This time, a specialized private company conducted the pest-control intervention over a 2-month period in the 56 rooms on the second floor (wings A and B); they treated furniture and clothing and placed silicone sealer around doors and floorboards to obstruct potential pest refuges. All furniture was removed, disassembled, and washed. When no bedbugs or eggs were found, bendiocarb was applied preventively; otherwise, curative *d*-*trans*-tetramethrin was applied (*3*). No further infestation has been observed.

Three pest-control interventions were required to eliminate these infestations. The first was not specific for bedbugs, and the second was not sufficiently extensive. Only specific and extensive insecticide application achieved elimination. The temporal–spatial distribution of dermatitis in this facility suggests 2 types of transmission: during the first 2 waves, spontaneous movement of the bedbugs is the most likely hypothesis because infested rooms were located near one another. During the last wave, bedbugs were most likely transported on clothing and/or furniture moved from room to room because affected rooms were 32 m from each other and no new resident had moved into the infested rooms or adjacent rooms (*3*).

Clusters of bedbug-infestation cases are well known in various communities, especially where living conditions are poor or in urban environments (*3*,*5*). This outbreak of bedbug dermatitis occurred in a nursing home. Because this type of outbreak in a medical facility can be considered healthcare associated, medicolegal implications must be considered and appropriate control measures adapted.

Increased worldwide travel (*9*) and insecticide resistance (*6*) contribute to the resurgence of bedbug dermatitis. Because the cockroach co-inhabits with bedbugs in the same biotope, as demonstrated by Émile Brumpt in 1936 (*10*), recent changes in pest-control techniques (i.e., use of selective cockroach-attracting traps that spare bedbugs) could be another factor enabling bedbug reemergence. At this time, healthcare facilities provide a welcoming environment for future bedbug-dermatitis outbreaks.
